# Dihydroartemisinin up‐regulates VE‐cadherin expression in human renal glomerular endothelial cells

**DOI:** 10.1111/jcmm.13448

**Published:** 2017-11-29

**Authors:** Liqun Li, Xiaocui Chen, Fengyun Dong, Qiang Liu, Caiqing Zhang, Dongmei Xu, Thaddeus D. Allen, Ju Liu

**Affiliations:** ^1^ Laboratory of Microvascular Medicine Medical Research Center Shandong Provincial Qianfoshan Hospital Shandong University Jinan Shandong China; ^2^ Department of Respiratory and Critical Care Medicine Shandong Provincial Qianfoshan Hospital Shandong, University Jinan Shandong China; ^3^ Department of Nephrology Shandong Provincial Qianfoshan Hospital Shandong University Jinan Shandong China; ^4^ Tradewind BioScience Daly City CA USA

**Keywords:** dihydroartemisinin, glomerular endothelial cells, VE‐cadherin, TGF‐β signalling

## Abstract

The antimalarial agent dihydroartemisinin (DHA) has been shown to be anti‐inflammatory. In this study, we found that DHA increased the expression of the junctional protein vascular endothelial (VE)‐cadherin in human renal glomerular endothelial cells. In addition, DHA inhibited TGF‐β RI‐Smad2/3 signalling and its downstream effectors SNAIL and SLUG, which repress *VE‐cadherin* gene transcription. Correspondingly, DHA decreased the binding of SNAIL and SLUG to the *VE‐cadherin* promoter. Together, our results suggest an effect of DHA in regulating glomerular permeability by elevation of VE‐cadherin expression.

## Introduction

In the kidney, vascular permeability is regulated by the glomerular filtration barrier (GFB), a highly specialized blood filtration interface maintaining the balance of ion and metabolite concentrations 1. The impairment of the GFB is the important feature of various renal inflammatory diseases 2. The GFB is composed of glomerular endothelium, the glomerular basement membrane (GBM) and the podocyte layer 3. The glomerular endothelium is a semipermeable membrane formed by glomerular endothelial cells (GECs), which are a unique microvascular cell type with round shape and fenestrations 2. GECs are exposed to circulating elements of the blood and are sensitive to various inflammatory factors 2. With dysfunction of the GFB, glomerular capillaries become highly permeable to water, solutes and plasma proteins, resulting in oedema and albuminuria 4. GECs are connected by adherens, tight and gap junctions, which maintain cell to cell adhesion and control vascular permeability 5. VE‐cadherin is expressed exclusively in endothelial cells and is a major component of vascular adherens junctions 6.

Artemisinin is a sesquiterpene lactone endoperoxide extracted from the Artemisia annua plant 7. It is widely used as an antimalarial drug due to its ability to inhibit the sarcoplasmic and endoplasmic reticulum calcium ATPase of Plasmodium falciparum 8. DHA is a water‐soluble derivative of artemisinin that produces few adverse side effects 8. Artemisinin and its derivatives displayed strong anti‐inflammatory effects 9. However, the underlying mechanisms have not been fully understood.

In this study, we evaluated the effects of DHA on the expression of VE‐cadherin in human renal glomerular endothelial cells (HRGECs). We found that DHA significantly elevated the expression of VE‐cadherin and inhibited transforming growth factor receptor I (TGF‐β RI)‐Smad2/3 signalling in HRGECs. In addition, DHA down‐regulated expression of SNAIL and SLUG, the transcriptional repressors of the *VE‐cadherin* gene. ChIP assay demonstrated that DHA significantly decreased the binding of SNAIL and SLUG to the *VE‐cadherin* promoter.

## Materials and methods

### Cell culture and treatments

HRGECs were obtained from Sciencell Research Laboratories (Carlsbad, CA, USA) and cultured in Dulbecco's modified Eagle's medium (DMEM) (Corning Inc., Corning, NY, USA), supplemented with 10% foetal bovine serum (Lonza, Basel, Switzerland), 100 IU/ml penicillin and 100 μg/ml streptomycin. DHA was purchased from Sigma‐Aldrich (St. Louis, MO, USA) and applied to HRGEC cultures with a final concentration 25 μM for 24 hrs before measurements.

### Western blotting

Western blotting was performed as previously described 10. The primary antibodies were rabbit anti‐VE‐cadherin, mouse anti‐SNAIL, rabbit anti‐SLUG, rabbit anti‐Smad2 and rabbit anti‐phospho‐Smad2 (pSer255) (Abcam, Cambridge, MA, USA), rabbit anti‐Smad3, rabbit anti‐phospho‐Smad3 (pSer423/425) and rabbit anti‐GAPDH (Cell Signaling Technology, Beverly, MA, USA) and rabbit anti‐TGF‐β RI (Santa Cruz Biotechnology, Santa Cruz, CA, USA). The secondary antibodies were HRP‐conjugated goat anti‐rabbit IgG and HRP‐conjugated goat antimouse IgG (Proteintech, Chicago, IL, USA).

### Quantitative real‐time PCR

Total cellular RNA was extracted from HRGECs with the E.Z.N.A. total RNA Kit II (OMEGA Bio‐tek, Inc., Norcross, GA, USA) following the manufacturer's protocol. Synthesis of cDNA was performed with the RevertAid First strand cDNA Synthesis kit (Thermo Fisher, Grand Island, NY, USA). QRT‐PCR was performed with a ViiA7 Real‐Time PCR System (Applied Biosystems, Waltham, MA, USA). Relative expression was calculated using β‐actin or GAPDH as an endogenous internal control. The primer sequences were listed in Table S1.

### Chromatin immunoprecipitation (ChIP) assay

Chromatin fragments of HRGECs were prepared as previously described 11. Immunoprecipitation was performed with a ChIP assay kit (Upstate Biotechnology Inc. Lake Placid, NY, USA) with the antibodies against SNAIL, SLUG or control IgG (Abcam) according to the manufacturer's instructions. The DNA fragments were detected by semi‐quantitative PCR. The primer sequences were as follows: sense, 5′‐GGGTGGACAAGCACCTTAAA‐3′; antisense, 5′‐ACCCCACTTGAACCCCTACT‐3′. The detailed materials and methods was described in Data S1.

## Results and discussion

### DHA increases the expression of VE‐cadherin in HRGECs

**Figure 1:**
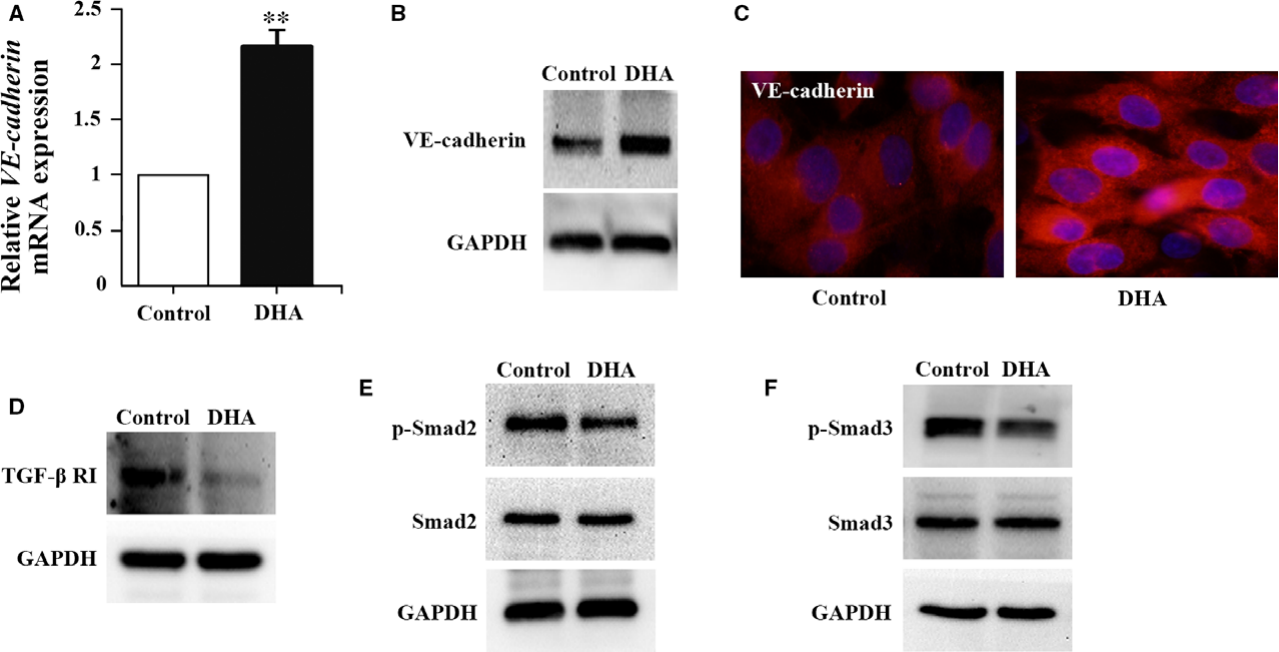
DHA up‐regulates the expression of VE‐cadherin and inhibits TGF‐β signalling in HRGECs. (**A**) Relative *VE‐cadherin* mRNA expression in HRGECs treated with vehicle or DHA (*n* = 4; **P <* *0.01). (**B**) Immunoblots of VE‐cadherin protein from HRGECs treated with vehicle or DHA. GAPDH was used as loading control. (**C**) Representative images of VE‐cadherin immunostaining on HRGECs treated with vehicle or DHA. Magnification: 200X. (**D**) Immunoblots of TGF‐β RI protein from HRGECs treated with vehicle or DHA. (**E**) Immunoblots of phospho‐Smad2 and total Smad2 from HRGECs treated with vehicle or DHA (**F**) Immunoblots of phospho‐Smad3 and total Smad3 from HRGECs treated with vehicle or DHA. GAPDH was used as loading control.

VE‐cadherin is a transmembrane adhesion molecule bridging adjacent endothelial cells 6. Inside cells, VE‐cadherin is complexed with β‐catenin and p120, which, in turn, bind to α‐catenin, an actin‐binding protein 12. The VE‐cadherin–catenin complex is essential for the maintenance of vascular integrity. The effect of DHA on the expression of VE‐cadherin in HRGECs has been examined. We found a significant up‐regulation of *VE‐cadherin* mRNA following 25 μM DHA treatments for 24 hrs (*P *<* *0.01, Fig. 1A). Consistently, VE‐cadherin protein was also increased after 24 hrs of DHA treatment (Fig. 1B). Immunofluorescent staining of HRGECs monolayers demonstrated that the intensity of VE‐cadherin plasma membrane staining was significantly increased in HRGECs after 24 hrs of DHA exposure (Fig. 1C). These results indicated that DHA increased VE‐cadherin expression in HRGECs. Up‐regulation of VE‐cadherin in HRGECs reduces vascular permeability 10. Therefore, it is likely that DHA‐induced up‐regulation of VE‐cadherin directly antagonizes glomerular hyperpermeability in renal inflammation diseases.

### DHA inhibits TGF‐β RI‐Smad2/3 signalling in HRGECs

Upon activation with TGF‐β, the TGF‐β RI induces a downstream signalling cascade that includes the phosphorylation of Smad2/3 13. In endothelial cells, phosphorylated Smad2/3 translocates into the nucleus and bind to the Smad‐binding element (SBE), activating the expression of target genes 13. We examined the effects of DHA treatment on the TGF‐β pathway in HRGECs. Western blot analysis showed that the expression of TGF‐β RI protein was decreased in HRGECs after incubation with DHA (Fig. 1D). Levels of phosphorylated Smad2 and phosphorylated Smad3 were also decreased following DHA treatment, whereas total levels of Smad2 and Smad3 remained unchanged (Fig. 1E and F). Previous studies showed that TGF‐β signalling impairs the barrier function of microvascular endothelial monolayers through down‐regulating the expression VE‐cadherin 11, 14. Thus, suppression of TGF‐β RI‐Smad2/3 signalling might contribute to DHA‐induced up‐regulation of VE‐cadherin.

### DHA suppresses the expression of SNAIL and SLUG in HRGECs

**Figure 2:**
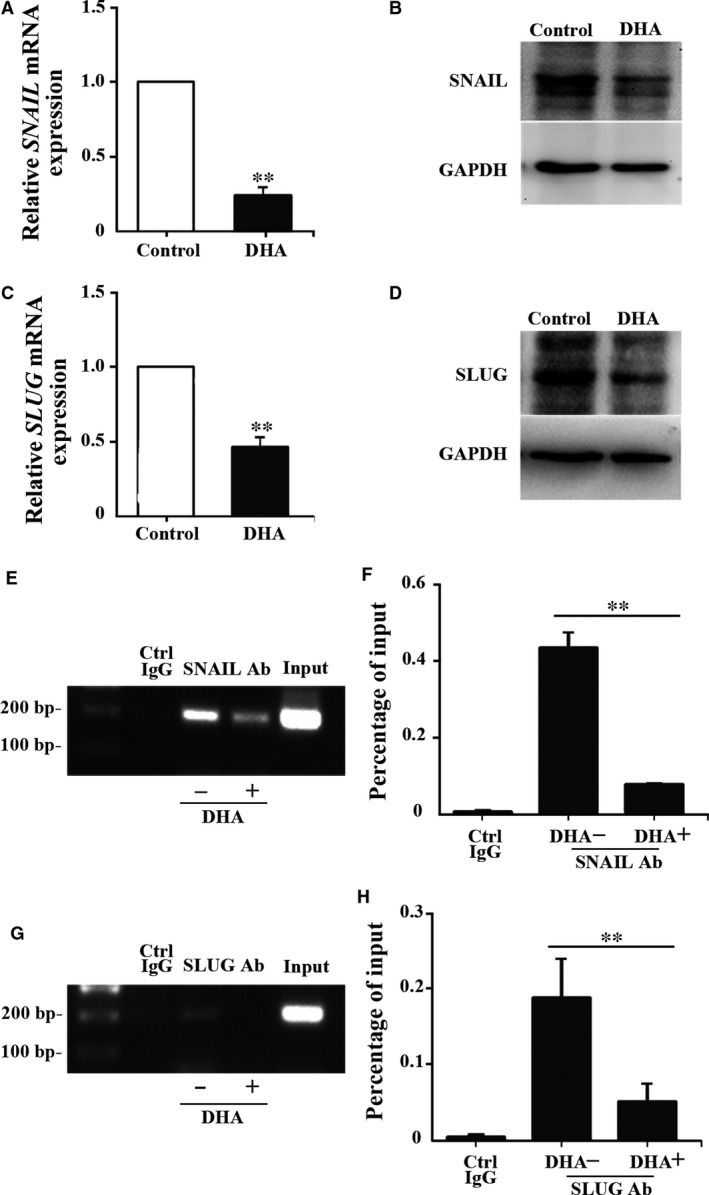
DHA inhibits the expression of SNAIL/SLUG in HRGECs and suppresses SNAIL/SLUG binding affinity to the human *VE‐cadherin* promoter**.** HRGECs were treated with 25 μM DHA for 24 hrs before measurements. (**A**,** C**) Relative mRNA expression of *SNAIL* (**A**) and *SLUG* (**C**) measured by qRT‐PCR in HRGECs treated with vehicle or DHA (*n* = 4; **P <* *0.01). (**B**,** D**) Immunoblots of SNAIL (**B**) and SLUG (**D**) protein from HRGECs treated with DHA. GAPDH was used as loading control. (**E**,** G**) Representative images of PCR products from the DNA fragments pulled down by SNAIL (**E**) and SLUG (**G**) antibodies. Primers were designed to detect the ‐240 E‐box on the human *VE‐cadherin* promoter. (**F**,** H**) Binding ratios relative to the total input of chromatin used for ChIP with the SNAIL (**F**) and SLUG (**H**) antibodies (*n* = 4; **P <* *0.01).

The SNAIL family of zinc‐finger transcription factors is known regulators of *VE‐cadherin* 15. *SNAIL* and *SLUG* are downstream effectors of TGF‐β RI‐Smad2/3 signalling 16. Smad proteins bind directly to the SBE of the *SNAIL* and *SLUG* promoters to elevate their transcription 16. Therefore, we examined the effect of DHA on SNAIL and SLUG expression in HRGECs. QRT‐PCR analysis demonstrated that DHA treatment markedly decreased the mRNA expression of *SNAIL* (*P *<* *0.01, Fig. 2A) and *SLUG* (*P *<* *0.01, Fig. 2C). Western blot analysis confirmed that both SNAIL and SLUG protein were decreased in HRGECs after DHA treatment (Fig. 2B and D). SNAIL and SLUG bind to the specific nucleotide sequence CANNTG, called the E‐box motif, through highly conserved C2H2‐type zinc‐finger domains 15. In endothelial cells, SNAIL and SLUG bind to the proximal E‐boxes (at ‐240) of human *VE‐cadherin* promoter and suppress its promoter activity 15. To examine the binding affinity of SNAIL and SLUG, ChIP assays were performed with HRGECs treated with DHA. Native chromatin was immunoprecipitated with antibodies raised against SNAIL, SLUG or control IgG, and the immunoprecipitated fragments were subjected to PCR using specific primers flanking the ‐240 SNAIL/SLUG binding site on the promoter of the human *VE‐cadherin* gene. The binding of both SNAIL (Fig. 2E and F) and SLUG (Fig. 2G and H) to the *VE‐cadherin* promoter was remarkably reduced by DHA treatment (*P *<* *0.01). This suggests that DHA increases the expression of VE‐cadherin *via* a cascade of events that include inhibitory effects on SNAIL and SLUG.

In conclusion, we found that DHA significantly increases the expression of VE‐cadherin in HRGECs. DHA inhibits TGF‐β RI‐Smad2/3 signalling and the downstream transcriptional activation of *SNAIL* and *SLUG*. In addition, DHA treatment decreased the binding of SNAIL and SLUG protein to the *VE‐cadherin* promoter. Together, DHA‐induced increase in VE‐cadherin expression arises through down‐regulation of basal levels of TGF‐β RI‐Smad2/3 signalling, lower expression of SNAIL and SLUG and ultimately a relief of transcription repression of the *VE‐cadherin* promoter.

## Conflict of interest

The authors declare no conflict of interest.

## Supporting information


**Table S1.** Quantitative RT‐PCR primer sequences

**Data S1.** Materials and Methods
