# Supplementary material for: Dihydroartemisinin up‐regulates VE‐cadherin expression in human renal glomerular endothelial cells
Source: J Cell Mol Med. 2017 Nov 29;22(3):2028–32. doi: 10.1111/jcmm.13448 (PMC5824371; doi:10.1111/jcmm.13448)
Supplement: Supplementary file 1 — Table S1. Quantitative RT‐PCR primer sequences [file JCMM-22-2028-s001.doc]

**Supplementary Table 1: Quantitative RT-PCR primer sequences**

| Gene | Sequence | Size, bp | Tm, ˚C |
| --- | --- | --- | --- |
| *VE-cadherin* |  |  |  |
| Sense | GCGACTACCAGGACGCTTTCA | 150 | 59.5 |
| Antisense | CATGTATCGGAGGTCGATGGTG |  |  |
| *SNAIL* |  |  |  |
| Sense | GAGGCGGTGGCAGACTAG | 178 | 59.5 |
| Antisense | GACACATCGGTCAGACCAG |  |  |
| *SLUG* |  |  |  |
| Sense | CATGCCTGTCATACCACAAC | 169 | 56.7 |
| Antisense | GGTGTCAGATGGAGGAGGG |  |  |
| *GAPDH* |  |  |  |
| Sense | TGATGACATCAAGAAGGTGGTGAAG | 240 | 57.9 |
| Antisense  *β‑actin*  Sense  Antisense | TCCTTGGAGGCCATGTGGGCCA  GGCACCACACCTTCTACAATG  GTGGTGGTGAAGCTGTAGCC | 352 | 59.1 |

All sequences are in the 5′–3′ orientation.
