# Supplementary material for: Dihydroartemisinin up‐regulates VE‐cadherin expression in human renal glomerular endothelial cells
Source: J Cell Mol Med. 2017 Nov 29;22(3):2028–32. doi: 10.1111/jcmm.13448 (PMC5824371; doi:10.1111/jcmm.13448)
Supplement: Supplementary file 2 — Data S1. Materials and Methods [file JCMM-22-2028-s002.doc]

**Supplementary Materials and Methods**

**Cell cultures and treatments**

HRGECs were purchased from ScienCell Research Laboratories (Carlsbad, CA, USA), and cultured in Dulbecco's modified Eagle's medium (DMEM) (Corning Inc., Corning, NY, USA), supplemented with 10% fetal bovine serum (Lonza, Basel, Switzerland), 100 IU/ml penicillin and 100 μg/ml streptomycin. The culture plates were incubated in humidified air at 37°C with 5% CO_2_. DHA was purchased from Sigma Aldrich (St. Louis, MO, USA) and applied to HRGECs cultures with a final concentration 25 μM for 24 hrs before measurements.

**Western blotting**

Total cellular protein extract was prepared from HRGECs. Protein concentration was determined using the BCA assay (Bio-Rad, Hercules, CA, USA). Equal amounts of protein (40 μg) were separated in a 10% polyacrylamide gel by SDS-PAGE, and transferred to a polyvinylidene ﬂuoride (PVDF) membrane (Sigma Aldrich). Then the membrane was blocked with 2.5% Bovine Serum Albumin (BSA) in Tris-buffered saline Tween (TBST) at room temperature for 2 hrs, and incubated overnight at 4°C with primary antibody. After washing 3 times with TBST, the membranes were incubated with HRP-conjugated secondary antibody (1:6000). Visualization was achieved using an ECL chemiluminescence kit (Millipore, Billerica, MA, USA). The primary antibodies were rabbit anti-VE-cadherin (33168), mouse anti-SNAIL (167609), rabbit anti-SLUG (106077), rabbit anti-Smad2 (40855) and rabbit anti-phospho-Smad2 (pSer255)(18834) (Abcam, Cambridge, MA, USA), rabbit anti-Smad3 (9513S), rabbit anti-phospho-Smad3 (pSer423/425)(9520S) and rabbit anti-GAPDH (2118) (Cell Signaling Technology, Beverly, MA, USA) and rabbit anti-TGF-β RI (Santa Cruz Biotechnology, Santa Cruz, CA, USA). The secondary antibodies were HRP-conjugated goat anti-rabbit IgG and HRP-conjugated goat anti-mouse IgG (Proteintech, Chicago, IL, USA).

**Immunofluorescence**

HRGECs were grown into monolayer on fibronectin-coated glass chamber slides and were then treated with 25 µM DHA for 24 hrs. The medium was aspirated, and the monolayers were washed with phosphate buffered saline (PBS), fixed with 4% paraformaldehyde, and washed 3 times with PBS for 15 min. Fixed cells were permeabilized with 0.1% Triton X-100 for 10 min and washed 3 times with PBS for 15 min. Cells were stained with a primary antibody against human VE-cadherin (Abcam, Cambridge, MA, USA) at a dilution of 1:500, overnight at 4°C and a rhodamine-labeled secondary antibody (1:200; Proteintech) for 30 min. Slides were imaged using an Olympus LCX100 Imaging System (Olympus Corporation, Tokyo, Japan) with an excitation wavelength of 546 nm.

**Quantitative real-time PCR**

Total cellular RNA was extracted from HRGECs with the E.Z.N.A. total RNA Kit II (OMEGA Bio-tek, Inc., Norcross, GA, USA) following the manufacturer’s protocol. Synthesis of cDNA was performed using the RevertAid First strand cDNA Synthesis kit (Thermo Fisher, Grand Island, NY, USA). QRT-PCR was performed using a ViiA7 Real-Time PCR System (Applied Biosystems, Waltham, MA, USA). Reaction conditions were 95°C for 15 min, 40 cycles of 95°C for 10 sec, and 60°C for 32 sec. All the PCR reactions were repeated in triplicate. Relative expression was calculated using β-actin or GAPDH as an endogenous internal control. The primer sequences were listed in supplementary Table 1.

**Chromatin immunoprecipitation (ChIP) assay**

HRGECs were exposed to 2% paraformaldehyde for 10 min and then treated with 0.2 M glycine for 5 min. The cells were washed twice with PBS containing protease inhibitor cocktail (Roche, Basel, Switzerland), then scraped and collected by centrifugation. The cell pellets were sonicated with a Branson 250 Sonifier to shear the chromatin into 300–700 bp fragments. Immunoprecipitation was performed using a ChIP assay kit (Upstate Biotechnology Inc. Lake Placid, NY, USA) according to the manufacturer’s instructions. The chromatin fragments were immunoprecipitated with the antibody against SNAIL, SLUG or control IgG (Abcam). The DNA fragments were detected by semi-quantitative PCR. The primer sequences were as follows: sense, 5’-GGGTGGACAAGCACCTTAAA-3’; antisense, 5’-ACCCCACTTGAACCCCTACT-3’.

**Statistical analysis**

Graphed data represent mean ± standard error. Statistical significance was evaluated using Student’s t-tests (two-tailed). All statistical analysis was performed using SPSS 17.0 software (SPSS Inc., Chicago, IL, USA). P < 0.05 was considered statistically significant.
